# Health related quality of life among Iraqi immigrants settled in Malaysia

**DOI:** 10.1186/1471-2458-11-407

**Published:** 2011-05-30

**Authors:** Aqil M Daher, Hisham S Ibrahim, Thaaer M Daher, Ali k Anbori

**Affiliations:** 1 Department of Population Health and Preventive Medicine, Faculty of Medicine, Universiti Teknology MARA(UiTM), Shah Alam, Selangor, Malaysia; 2 Department of Physiology, Faculty of Medicine, Universiti Teknology MARA(UiTM), Shah Alam, Selangor, Malaysia; 3 Department of Surgery, Faculty of Medicine, Management and Science University(MSU), Shah Alam, Selangor, Malaysia; 4 Department of Social and Preventive Medicine, Faculty of Medicine, University Malaya (UM), Kuala Lumpur, Malaysia

## Abstract

**Background:**

Migrants everywhere face several demands for health and maintaining good health and quality of life could be challenging. Iraqis are the second largest refugee group that has sought refuge in the recent years, yet little is known about their health related quality of life (HRQOL). The study aims at assessing the HRQOL among Iraqis living in Malaysia.

**Methods:**

A self-administered Arabic version of Sf-36 questionnaire was distributed among 300 Iraqi migrants in Malaysia. The questionnaire taps eight concepts of physical and mental health to assess the HRQOL. Univariate analysis was performed for group analysis (t test, ANOVA) and Multiple Linear Regression was used to control for confounding effects.

**Results:**

Two hundred and fifty three participants ranging in age from 18 to 67 years (Mean = 33.6) returned the completed questionnaire. The majority was males (60.1%) and more than half of the respondents (59.5%) were married. Less than half (45.4%) and about a quarter (25.9%) reported bachelor degree and secondary school education respectively and the remaining 28.7% had either a master or a PhD degree.

Univariate analysis showed that the HRQOL scores among male immigrants were found to be higher than those of females in physical function (80.0 vs. 73.5), general health (72.5 vs. 60.7) and bodily pain (87.9 vs. 72.5) subscales. The youngest age group had significantly higher physical function (79.32) and lower mental health scores (57.62).

The mean score of physical component summary was higher than the mental component summary mean score (70.22 vs. 63.34).

Stepwise multiple linear regression, revealed that gender was significantly associated with physical component summary (β = - 6.06, p = 0.007) and marital status was associated with mental component summary (β = 7.08, p = 0.003).

**Conclusions:**

From the data it appears that Iraqi immigrants living in Malaysia have HRQOL scores that might be considered to indicate a relatively moderate HRQOL. The HRQOL is significantly affected by gender and marital status. Further studies are needed to explore determinants of HRQOL consequent to immigration. The findings could be worthy of further exploration.

## Background

Human migration has been described as 'one of the defining issues of the 21st century'. It refers to the movement of a person or group of people from one geographical region to another, across an administrative or a political border, to settle definitively or temporarily [1]. Various types and practices of migration have been identified including orderly migration, return migration, forced migration, irregular migration, smuggling, and trafficking. Migration takes place mainly because of the outbreak, renewal and prolongation of armed conflict has a negative impact on peace, stability and security in many regions of the world [2].

Among the 42 million forcibly displaced people worldwide at the end of 2008, Iraqis form the second largest refugee group, with 1.9 million having sought refuge mainly in neighboring countries [3]. Despite the fact that Iraq is one of the largest oil producing countries in the world, many Iraqis have been displaced from their country mainly for political reasons prior to American invasion of Iraq in 2003 and thereafter due to loss of security, the collapse of the infrastructure and political and sectarian violence [4].

In view of the rapidly growing global mobility, migration health has become an important field within the field of medical sciences[5]. Measuring health status of a population is not just limited to the traditional measures of morbidity and mortality, but also the assessment of Quality Of Life (QOL). QOL is a multi-dimensional concept, which encompasses crucial areas such as physical health, psychological well being, social relationships, economic circumstances, personal beliefs and their relationships to salient features of the environment. Health-Related Quality of Life (HRQOL) refers to the patient's sense of his own health and well-being in the broad areas of physical, psychological and social functioning[6].

Migrants face several challenges for health, among those include income and educational achievements. Exposure to new lifestyles may affect both physical and mental health [7,8], and language gap, when present, together with lack of proper health insurance cover could significantly undermine access to health care [9]. Racial discrimination, when present, can also result in inequalities in the provision of health care and could adversely impact opportunities in and quality of health care[10]. Lack of cohesive social support and cultural gaps may influence health behavior [11]. As such, maintaining good health and quality of life can be very challenging to a lot of the migrants.

Migrants in Spain have been reported to have worse HRQOL than the Spanish natives, which is incidental to their disadvantages in socioeconomic status, social support, and psychological distress[12]. Although the mental component of HRQOL among Mexican Americans living in colonias was of similar status when compared to the general population of the United States, the physical health component was however significantly worse. Poor education and long-term residency in colonias were predictors of lower physical health[13].

A group of elderly Iranians in Sweden reported a HRQOL worse than that of Swedish citizens [14]. Migration was also reported to affect negatively the sleep quality of some Portuguese and Moroccan women when compared to German women [15]. It has been suggested that discrepancy between aspiration and achievement, which can result in poor self-esteem, leads to depression among immigrants [16]. The impact of migration might also to depend on differences in the developed status between the migrant's country of origin and the country of eventual residence. For example, migrants from Germany and France to Switzerland have reported that their status of health did not differ significantly from that of the majority of the Swiss population, while immigrants from Italy, the former Yugoslavia, Portugal, Spain and Turkey, on the other hand reported lower levels of health status [17].

Findings from Germany have also indicated that inadequate medical care due to language barriers or sociocultural differences leads to a higher rate of chronic headache among first generation of Turkish immigrants in Germany [18].

Immigrants who reported financial and job strain, suffer greater impact from their pain conditions and exhibit higher levels of emotional distress, compared to natives[15,19].

On the other hand, migration may contribute to a better QOL of immigrants compared to that of their countrymen, especially when immigration takes place into a country with higher human development index and better amenities and accessibility to health care services [14].

Latino immigrants in the USA reported that exposure to political violence was higher in their country of origin, which was found to be a predictor of associated impairments in mental health and health-related quality of life [20].

The quality of life among a group of Turkish immigrants in Sweden was found to be better when compared with the Turkish people [21].

Most information about immigration and health has come from studies carried out in the developed countries where refugees sought a new home. These studies have mainly emphasized on the predictors of HRQOL and sought a comparison with the native population to determine the effect of immigration on HRQOL.

Very few studies have actually documented information on the health of migrants to less developed or developing countries. In this regard, there is very little research carried out on the health aspects of Iraqi immigrant population and there is an extreme paucity of literature on the subject. We strongly felt it as a necessity to prepare a database of the Iraqi immigrants in Malaysia, which would be of use in future evaluation.

Understanding HRQOL is critical in order to track and improve the health of the poor, vulnerable population to reduce the health care disparities. The aim of this study is therefore to assess the HRQOL among a sample of Iraqis living in Malaysia

## Methods

### Sample size calculation

We estimated that 252 subjects are adequate to detect a mean difference of 5 in any subscales of SF 36 with standard deviation of 21 at alpha of 0.05 with power of 0.8.

The sample size was increased up to 300 to allow for non response.

### Sampling method

Participants were selected by simple random sampling using Epi Info 6 software from a sampling frame consisting of a list of registered immigrants with their residence and email addresses obtained from the Iraqi embassy.

### Participants

This cross sectional study was conducted between September 2009 and April 2010 in which we recruited a sample of 300 Iraqi immigrants in Malaysia. Criteria for inclusion were: Adult aged 18 and above and able to read and write Arabic.

The participants were contacted personally and were informed about the aims of the study and that the participation is voluntary. Also they were informed that the results of the study will be used for publication and no personal data will be revealed. Questionnaires were only distributed to those who had consented to participate in the study. There were 47 subjects who did not respond to the questionnaire. The analysis of their characteristic showed that there was no significant difference from the respondents except in regard to gender where the proportion of males who did not respond was slightly higher than females, but still not significant.

### Instrument and procedures

A translated Arabic version of the MOS 36-Item Short Form Health Survey (SF-36) was used in this study [22]. This questionnaire is widely used in many countries and has been translated into many languages and proved to be valid and reliable.

The Arabic version was used in Arabic speaking countries where certain items were modified in order to fit more closely into the Arabic context, and consistent with the inherent norms of Arabic societies, which share almost the same socio-cultural norms [23,24].

The SF-36 Health Survey contains 36 items that taps eight health concepts: physical functioning (PF), role limitations due to physical health problems (REP), bodily pain (BP), general health (GH), vitality (VT), social functioning (SF), role limitations due to emotional problems (REE) and mental health (MH).

Majority of the participants were given the questionnaire in person and those living in distant places, the questionnaire was delivered by email. The questionnaire was self-administered and participants were also requested to provide information relating to their age, sex, occupation, marital status and educational level.

### Statistical analyses

All data were entered and analyzed using SPSS version 16. Descriptive statistics were calculated for all variables. Numerical variables were summarized as a mean ±SD and categorical variables summarized by frequency and percentage.

Independent samples t test was used to compare the mean between two groups and ANOVA for comparing the mean of more than two groups. Multiple Linear Regression (MLR) was performed to control for confounding variables. Significance level was set at 0.05.

### Scoring of SF 36

The SF 36 was scored according to the recommendation by Ware JE [25].

The SF-36 items describing the eight health concepts were transformed into a score of 0 -100 and the items scale averaged to obtain a subscale score. Physical Component Summary (PCS) and Mental Component Summary (MCS) were computed by averaging the values of the respective subscales. A higher score indicates higher levels of function and better health.

### Ethical Approval

This study was approved by the research ethics committee of Management and Sciences University (MSU).

## Results

The demographic characteristics of participants are presented in Table 1. A total of 253 Iraqi immigrants responded to the questionnaire. The age ranged from18-67 years with mean of 33.6 ± 12.2 years. Further categorization of age revealed that around half of the participants are below the age of 30 years, 22.5% between 30-39 years and the rest were above the age of 40 years. Majority of the respondents were males (60.1%) who also reported higher educational level and employment status. More than half of the respondents were married (59.5%). In terms of their education, 45.4% and about 25.9% reported holding a bachelor's degree and having secondary school education respectively and the remaining 28.7% had either a master or a PhD degree.

**Table 1:** Sociodemographic characteristics of study sample

Variable	Mean(SD)	N (%)
**Age **(years)	33.6(12.2)	
18-29		117(46.2)
30-39		57(22.5)
40-49		49(19.2)
50 +		30(11.9)
		
**Gender**		
Male		152 (60.1)
Female		101(39.9)
		
**Working status**		
Employed		74(29.7)
Student		120(48.2)
Housewife		48(19.3)
Unemployed		7(2.8)
		
**Marital Status**		
Single/Divorced		102(40.5)
Married		150(59.5)
		
**Highest Education attained**		
Primary school		0
Secondary		645(25.9)
Bachelor Degree		114(45.4)
Master		45(17.9)
PhD		27 (10.8)

The mean score of sf-36 scales for participants is presented in Table 2. PF was the highest among the 8 scales (77.4 ± 21.5) and VT was the lowest (56.8 ± 17.7). The physical component summary score was higher than the mental component summary score (70.22 ± 18.64 vs. 63.34 ± 18.52).

**Table 2:** Descriptive data on the SF-36 mean scores for each subscale by gender, age, education, marital, and working status.

	Age (years)					Gender				Marital Status	
Scales	18-29	30-39	40-49	50 +	*P value*	Male	Female	*P value*	Single/Divorced	Married	*P value*
PF	79.32(20.99)	78.23(22.96)	76.33(19.71)	67.86(21.44)	0.025*	80.0(20.4)	73.5(22.6)	0.018*	77.3(22.8)	77.6(20.7)	0.785
REP	67.83(35.93)	70.61(37.54)	68.88(35.18)	65.83(36.83)	*0.159*	70.3(36.2)	65.6()35.9	0.308	68.3(36.6)	68.5(35.9)	0.995
REE	57.75(38.16)	63.69 (40.83)	68.70 (39.91)	64.36 (41.72)	*0.105*	60.4(39.2)	64.4(40.1)	0.439	53.9(39.3)	67.3(38.9)	0.008*
VT	56.42(16.09)	58.30 (20.76)	56.83 (18.22)	55.16 (17.19)	*0.411*	57.9(17.7)	55.0(17.6)	0.193	55.7(16.4)	57.3(18.4)	0.485
MH	57.62(17.27)	65.35(17.12)	63.26(18.28)	69.46(17.08)	0.001*	60.9(17.8)	63.3(17.9)	0.301	56.4(18.4)	65.3(17.9)	0.001*
SF	71.76(20.19)	73.66(22.69)	77.29(18.16)	71.25(23.70)	*0.367*	71.9(21.2)	75.2(20.2)	0.207	71.2(20.9)	74.4(20.8)	0.24
BP	77.09(21.14)	76.91(22.72)	77.90(19.48)	70.25(21.71)	*0.664*	78.9(20.3)	72.5(22.2)	0.018	78.1(21.4)	75.3(21.2)	0.3
GH	59.82(12.87)	62.45(18.80)	56.32(19.19)	54.48(16.86)	*0.065*	72.5(22.2)	60.7(16.2)	0.04*	61.1(14.0)	57.8(17.6)	0.128
PCS	70.97(16.80)	72.46(20.91)	69.85(18.67)	63.88(20.49)	0.238	72.67(17.77)	66.70(19.42)	.014*	69.42(19.05)	71.48(18.13)	.401
MCS	60.73(16.90)	65.59(20.12)	66.52(18.31)	64.85(20.90)	0.191	62.83(18.80)	64.32(18.05)	.535	59.10(17.67)	66.18(18.43)	.003*
	**Working status**				**Highest Education Attained**						
Scale	Employed	Student	Housewife	Unemployed	*P value*		Secondary	Bachelor Degree	Master	PhD	*P value*
PF	80.1 (19.7)	77.3 (22.1)	76.6(20.9)	55.7(22.6)	0.038*		73.2(25.9)	78.8 (18.5)	77.4 (23.4)	81.2(17.9)	0.293
REP	68.6(38.2)	69.1(35.9)	67.2(34.3)	60.7(37.8)	0.939		62.5 (37.0)	68.6(35.9)	76.1(35.7)	67.6(35.2)	0.289
REE	64.8(40.7)	57.4 (39.6)	66.7(39.7)	76.2(37.0)	0.32		58.9(38.9)	62.8 (39.8)	64.4(39.6)	60.2 (42.2)	0.89
VT	57.8(18.6)	56.3(16.7)	56.5(20.0)	53.6(9.9)	0.906		55.0 (16.5)	56.1 (17.6)	58.6 (17.5)	60.4 (21.4)	0.496
MH	65.23(16.5)	58.5 (17.1)	62.7(20.0)	73.7(19.7)	0.017*		57.2(18.2)	61.6 (18.7)	66.3 (141)	65.8(17.5)	0.036*
SF	72.6(20.9)	72.2 (20.6)	75.3(21.3)	78.6(20.0)	0.746		72.1(21.7)	73.7(20.6)	74.2(18.2)	71.3(24.9)	0.907
BP	74.1(21.8)	79.7 (20.4)	71.6(21.8)	73.6(24.4)	0.093		73.0 (22.2)	75.8 (22.2)	81.3(18.1)	77.9(19.3)	0.239
GH	59.0(17.6)	60.9(14.8)	55.7(17.94)	55.0(9.6)	0.283		59.3(15.9)	57.9 (15.6)	60.7(16.8)	61.7(19.3)	0.649
PCS	70.23(18.92)	71.90(18.04)	67.47(19.32)	61.25(20.92)	0.314		67.17(20.25)	70.10(18.06)	73.87(17.70)	71.56(19.09)	0.325
MCS	65.30(19.59)	60.93(17.65)	65.26(18.84)	70.51(17.80)	0.228		60.54(17.06)	63.56(18.72)	66.33(17.19)	64.20(22.92)	0.445

* *p *value significant at 0.05

Table 3 summarizes the association between the sociodemographic characteristics of respondents and their HRQOL. Only age was significantly associated with PF (p = 0.025) and MH (p = 0.001), where the highest PF scores (79.32 ± 20.99) and the lowest MH scores (57.62 ± 17.27) were in the youngest age group.

**Table 3:** Mean scores of HRQOL of Iraqi immigrants

Scales	Mean(SD)
Physical functioning (PF)	77.4(21.5)
Role physical(REP)	68.4(36.1)
Role emotional (REE)	62(39.5)
Vitality (VT)	56.8(17.7)
Mental health (MH)	61.9(17.8)
Social functioning (SF)	73.2 (20.9)
Bodily pain (BP)	76.4(21.2)
General health (GH)	59.1(16.3)
Physical Component Summary (PCS)	70.22(18.64)
Mental Component Summary (MCS)	63.34(18.52)

Women had significantly lower scores than men in three of the physical components, namely; PF (p = 0.018), GH (p = 0.04) and BP (p = 0.018) and overall lower PCS (p = 0.014).

Although participants with lower education had lower scores in most of the eight subscales, only MH was significantly associated with education (p = 0.036).

Those in employment had significantly higher PF score (p = 0.038) whereas the unemployed had the highest MH score (p = 0.017) compared to other work groups. Regarding marital status, it was found that those who were married had high scores in REE (p = 0.008), MH (p = 0.001) and MCS (p = 0.008).

Table 4 depicts the results of stepwise MLR, it is apparent that only gender is significantly associated with PCS (p = 0.007) and marital status associated with MCS (p = 0.004).

**Table 4:** Stepwise MLR for PCS and MCS of Iraqi sample controlling for confounding variables

	PCS				MCS			
Variable	B	SE	t	p	B	SE	t	P
Sex	- 6.06	2.42	-2.50	.001	-	-	-	-
Marital status	-	-	-	-	7.085	2.35	3.014	.003

Reference categories, male and single/divorced

## Discussion

To our knowledge, this is the first study assessing HRQOL among the Iraqi community residing outside Iraq. The major objective of this study was to determine the perception of the Iraqi immigrants about their quality of life within the Malaysian context. Quality of life as a measurement can identify groups with physical or mental health problems and provide a guide to intervention and follow-up evaluation.

It might be argued that the two different methods used to distribute the questionnaire might have influenced the results as clarification could be better sought by the respondents when the questionnaire is distributed by hand than by email. However, this was not the case as recipients in both situations had equal access to the researcher and generally there was very little clarification sought by the respondents. For both these methods, answering the questionnaire nevertheless imposed cognitive demands on the respondents equally, whereby the respondent had to have reading and comprehension skills and be literate in reading the language of the questionnaire.

Electronic methods required easy access to a computer and internet facilities, basic computer literacy, and also familiarity with numbers and keyboards. We made all necessary modifications to make the electronic word version of the questionnaire easily answerable [26]. On the whole we believe that the method of data collection did not have any significant effect on the results.

The reason for the majority of the respondents being young (18-29 years), is that this age group constitutes around 2.8 million of the total 31 million Iraqi population [27], and the age group that is most likely to migrate. Most of the Iraqis settled in Malaysia came in pursuit of their studies and to escape the unstable political condition in their home country. A few of the respondents were senior migrants who worked as professionals, mainly in the field of education.

Analysis of the relationship between the sociodemographic variables and HRQOL was inconclusive across the 8 scales. The finding that the youngest age is significantly associated with better PF and worse MH may be explained by the younger age-group's more active lifestyle compared to older age group [14,21] and having lower morbidity. Lower MH may be explained by the fact that the majority of the young were not married, which is found to be a contributing factor for better mental health [28] as also their position in the society, exposure to new culture, lifestyle and challenges. Similar findings have been reported among the Lebanese and Malaysian population using the same instrument where the older age had lower PF and higher MH [23,29].

Apart from MH, the non significant differences of the different scales observed among different educational levels may be explained by the fact that the Iraqi community has almost the same living condition, lifestyle and socio-cultural values including health perception [30]. Interestingly all the subjects in the study had secondary to higher and tertiary education and none with just primary or no formal education at all. This is not surprising given that education is compulsory in Iraq and most have attended school at least until the American invasion in 2003 [31].

The final MLR model showed that significant association is only present between gender and PCS and between marital status and MCS.

Differences in PCS with regard to gender could be explained mainly in terms of social perspectives and longevity. It has been documented that women live longer than men but experience more health problems. Men and women differ in their social behavior that prompts or avoids health problems and the way they adopt some risk behaviors and exposures [32]. Women usually report higher levels of health problems because they react differently than men to the material, behavioral and psychosocial conditions that foster health [33]. Females tend to be less employed compared to men in addition to inequalities in income and education [34]. The lower score among the females may also be explained by the hormonal variation, child birth and role as a mother and wife, these factors are often found to be the detrimental to a woman's health [35,36].

Iraqi females in our sample reported lower education level, higher marital status and lower employment. Similar findings were reported from studies on the Malaysian and Lebanese populations, where females scored lower than males in the mentioned QOL scales [23,29]. Similar results were reported from elderly Iranian population, where females had PCS and MCS worse than males[37]. Among a sample of Turkish immigrants, the quality of life of male immigrants was found to be higher than that of females [21].

Contradictory to our findings, a researcher using different QOL instrument reported that female Iranian refugees who were resettled in Sweden had the overall QOL and general health scores that were higher than those of Iranian males [38].

The significant difference in MH in regard to marital status may be attributed to stability and emotional support provided by the spouse. Marriage may provide an emotionally fulfilling, intimate relationship, satisfying the need for social connection, which could have implications on both physical and mental health[39]. In addition, a spouse may play an important role in monitoring and encouraging healthy

behaviors, such as good eating habits and regular exercise, as well as discouraging unhealthy ones, such as smoking or heavy drinking [40]. The most recent research examining the effect of marriage on health suggests that marriage reduces depressive symptoms for both men and women [41,42].

Marriage has been found to contribute to better physical and mental health. Married couples, who have two incomes would enjoy economic well-being which in turn could improve health outcomes by enhancing access to health care and lowering stress [43]. Married respondents in our sample had a higher level of education, which is conducive to better coping skills and access to health information. High education also contributes to a higher employability which in turn contributes to better financial stability. A group of married Turkish immigrants reported significant QOL difference from single and widowed immigrants[21].

In spite of the absence of objective evidence about the effect of the new environment, the data from this study suggests that Iraqi migrants in Malaysia have a relatively moderate QOL and enjoy the same living conditions as the Malaysians. They have an almost equal access to health care as the Malaysians, though they may incur some additional costs unless they are employed by the Malaysian Government.

Although the data has provided a first database of its kind of Iraqis living in Malaysia, there are however a number of limitations evident both in the study and its interpretation. Firstly, the lack of studies of HRQOL in the Iraqi population in Iraq limited the comparison between Iraqi immigrants in Malaysia with Iraqis living in Iraq.

Secondly, data on chronic illnesses and smoking habits among Iraqi immigrants were not collected because incorrect or under reporting of such variables might compromise the results.

Thirdly, generalization of the result might have been affected by the exclusion of those who were not registered with the Iraqi embassy.

Lastly a cross sectional study does not reflect change in HRQOL unless repeated at a different point of time.

## Conclusions

It appears that Iraqi immigrants in Malaysia have HRQOL scores that might be considered to indicate a relatively moderate HRQOL, which like in other studies, is influenced by gender and marital status. We hope that this study provokes the interest of researcher inside Iraq to assess the HRQOL among the Iraqi population, which will be of great value as no published data are so far available.

Without the availability of data from Iraq it is not possible to determine if the migration to Malaysia has had a positive or negative impact on the HRQOL of Iraqi immigrants.

Further studies assessing the factors affecting quality of life among Iraqi migrants are needed. We recommend future studies to include other factors that play a role in HRQOL of migrants, especially ethnic differences, coping with new environment and job and financial security.

## Competing interests

The authors declare that they have no competing interests.

## Authors' contributions

AMD conceived the study, collected the data and performed statistical analysis and drafted the manuscript. HSI participated to conceive and design the study, collected the data and helped to draft the manuscript. TMD and AKA participated in data collection and manuscript drafting. All authors have read and approved the final manuscript.

## Pre-publication history

The pre-publication history for this paper can be accessed here:

http://www.biomedcentral.com/1471-2458/11/407/prepub
